# Autotransplantation of Ectopic Permanent Maxillary Incisors

**DOI:** 10.1155/2017/7361924

**Published:** 2017-03-02

**Authors:** Sarimah Mohd Mokhtar, Laila Abd Jalil, Nurhidayah Muhd Noor

**Affiliations:** Department of Paediatric Dentistry, Tuanku Ja'afar Hospital Seremban, Ministry of Health of Malaysia, Negeri Sembilan, Malaysia

## Abstract

The report presents examples of successful cases of autotransplantation of ectopic teeth as donor in the treatment of clinically missing maxillary anterior teeth in young patients. The transplanted teeth were either severely ectopic, inverted, rotated or in an unfavourable position that they are commonly sacrificed as a result. Details of surgical technique as well as clinical and radiographic assessments were discussed.

## 1. Introduction

Conventional treatment plan for ectopically positioned tooth is surgical exposure and orthodontic treatment [1]. In severely rotated or inverted ectopic tooth where orthodontic traction is not possible, surgical removal and replacement with prosthesis are commonly opted. Autotransplantation in many cases is the only alternative to prosthetic substitution and is seldom considered as a treatment option in our country. Autotransplantation is our method of preference based on successful outcome of few reports [2–4]. Autogenous transplantation or autotransplantation is defined as the transplantation of embedded, impacted, or erupted teeth from one site into extraction sites or into surgically prepared sockets in the same person [5]. Included in this definition is intra-alveolar transplantation which was defined by Tsukiboshi as repositioning of a tooth in its own alveolus, such as in verticalization or surgical extrusion of a tooth [2]. This report presents three cases of intra-alveolar transplantation of ectopically positioned permanent maxillary incisors in young patients as an example of solutions to the problem of disrupted or delayed eruption of permanent maxillary incisors.

## 2. Presentation of Cases

**Figure 1:**
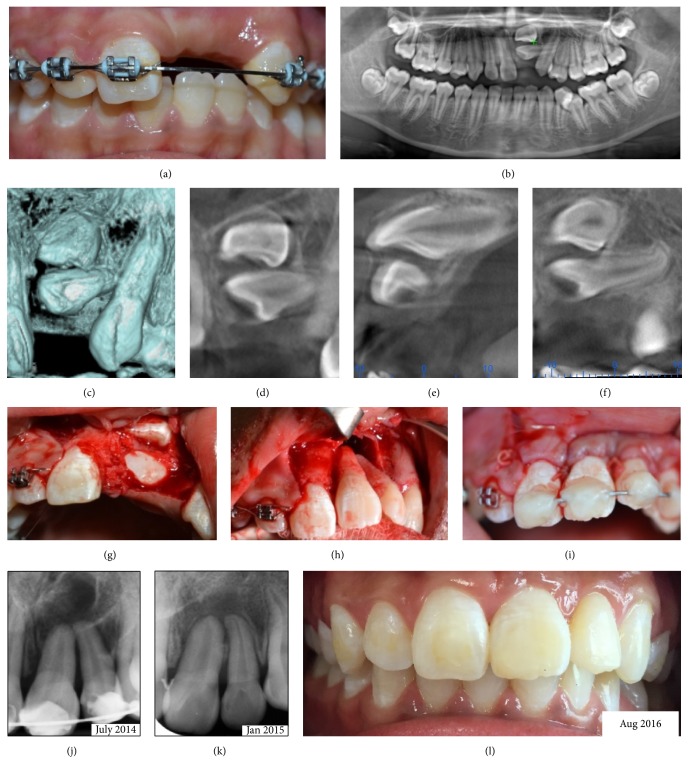


*Case 1*. A 14-year-old boy presented with unerupted upper left central incisor, 21, and upper left lateral incisor, 22, with space loss. Radiographical investigation using orthopantomogram (OPG) and cone beam computed tomography (CBCT) revealed that the 21 and 22 were horizontally impacted (Figures 1(c), 1(d), 1(e), and 1(f)). The treatment plan was for upper fixed appliance to open space followed by surgical autotransplantation of both 21 and 22. Once the space was adequate, surgery was carried out under general anaesthesia (Figures 1(a), 1(g), and 1(h)). Composite wire splinting was placed for 5 weeks (Figure 1(i)). As early as 6 months, complete bone deposition and presence of lamina dura were observed radiographically (Figure 1(k)). Clinically, there was no sign and symptom and both teeth had retained vitality at 2-year review (Figure 1(l)). 

**Figure 2:**
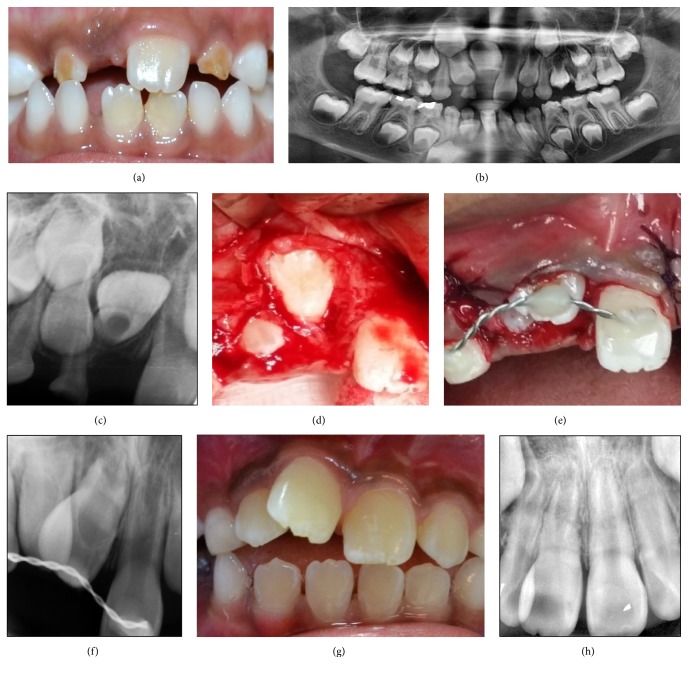


*Case 2*. A 7-year-old boy presented with unerupted upper right central incisor, 11, with history of multiple trauma on front teeth few years earlier (Figure 2(a)). Radiographical investigation carried out by OPG and periapical radiograph revealed an inverted and newly formed root of 11 (Figures 2(b) and 2(c)). Tooth 11 was manipulated gently and rotated 180 degrees to an acceptable position (Figures 2(d), 2(e), and 2(f)). The tooth was splinted with composite wire splinting for 4 weeks. At 6-week review, the tooth showed no sign and symptom and since then, it has continued to erupt (Figure 2(g)). Radiographically, at one year review, there was complete bone deposition, continuation of root growth, and presence of lamina dura (Figure 2(h)).

**Figure 3:**
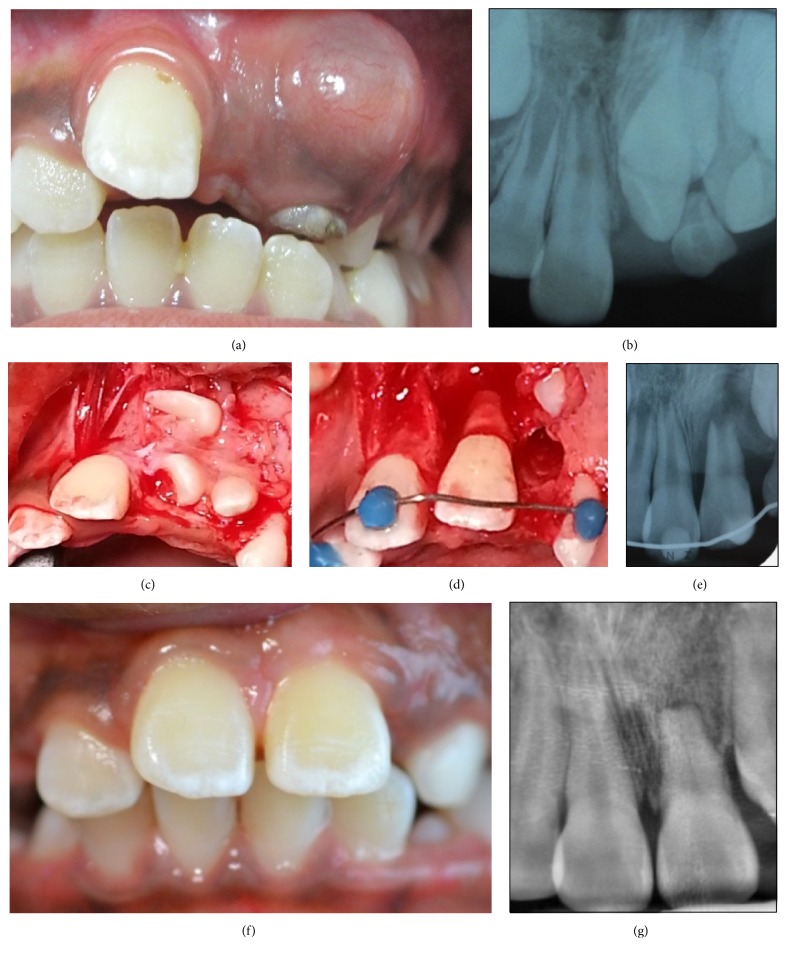


*Case 3*. A 9-year-old boy presented with delayed eruption of upper left central incisor, 21, with associated swelling and a retained carious deciduous tooth, 62 (Figure 3(a)). Radiographic examination revealed the presence of a supernumerary at the upper left lateral incisor, 22, and ectopic impaction of 21 and 22 (Figure 3(b)). Extraction of 62 was executed and brownish straw coloured fluid was discharged from the socket and the swelling immediately subsided. Cystic lining was sent for histopathological examination which later revealed a radicular cyst. Autotransplantation of 21 was carried out under general anaesthesia together with removal of the supernumerary and 22 (Figures 3(c), 3(d), and 3(e)). Tip of canine was noted preventing transplantation of 22. Bone graft materials are unnecessary between bony walls and transplanted root even if the space is wide [2] as was shown in this case.

The tooth was stabilized with composite wire splinting for 4 weeks. At 2-year review, 21 has adjusted itself into occlusal line (Figure 3(f)). It was positive on vitality testing, nontender to percussion, with no mobility and normal periodontal pocket. Radiographically, there was evidence of alveolar bone deposition, thickening of dentinal wall, continuation of root growth, and presence of lamina dura. However, the apical growth has stopped before full length and the pulp became obliterated as early as 6 months (Figure 3(g)). It was noted that the colour of the crown was more yellow than contralateral tooth.

### 2.1. Technique

Thorough clinical and radiographic assessments are essential prior to the surgery. Assessments were specifically carried out for buccolingual and mesiodistal width of both donor and recipient sites, root length and stage of root development of donor, position of the tooth and its immediate associated structures, and anticipation for any surgical difficulties. In cases where the mesio-distal width is not sufficient, space should be created by orthodontic treatment prior to surgical autotransplantation. Obtaining an informed consent is an essential part of patient management. Lengthy explanation of the procedures, risks and benefits, possibility of failure, and alternative treatment was ensured to the patient or parents.

The surgical technique was adapted from Tsukiboshi [2] and Andreasen et al. [6]. An aseptic technique was maintained throughout the procedures. The surgical site and surrounding perimeter were cleaned thoroughly with 0.2% chlorhexidine gluconate. Local anaesthetic was administered at the buccal and lingual sites with Scandonest 2% containing adrenaline 1 : 80000. Mucoperiosteal flap was raised and retracted.

The donor tooth was surgically loosened carefully from its original socket. It is critical that the periodontal ligament (PDL) cells are preserved and not damaged during surgery. The removed donor tooth must be stored in a moist and physiologic medium such as saline to maintain vital PDL cells. It would be better, and if possible, that the removed donor tooth is replaced back and kept in its socket while surgical preparation is carried out.

Estimation of recipient size was made well in advance during preoperative assessment and also during surgery. It is desirable that the width and length of available bone at the recipient site approximate to or are slightly larger than the size of donor. This is to ensure minimal trauma to the PDL during positioning of donor tooth onto the prepared site. During the surgery, with the aid of radiograph or CBCT, the recipient site was prepared using surgical round burs with copious saline irrigation. An attempt of fitting the donor to the recipient site was made swiftly. Minor surgical preparation adjustment sometimes was necessary for the final placement. The donor was then transplanted to the optimal possible position, taking extra precaution to maintain the root within alveolar bone. In most cases, the transplanted teeth are set in infraocclusion, free of any interference from the opposing teeth. It is the author's experience that autotransplantation of ectopic permanent incisor teeth requires less challenging surgical preparation provided that adequate preoperative assessment was performed. Also, it is quite common that removal of the ectopic donor leaves ample space for the fitting of the root of donor, leaving minor surgical preparation for the width of the crown.

A flexible composite wire (preferably twisted 0.5 stainless steel wire) splinting was placed to secure the transplant. Flexible splinting allows physiological movement of the transplanted tooth within the new bed which is important in osseoinduction and healing process. Splinting was removed between 2 and 8 weeks when the mobility has reduced to almost grade 1. It was our concern that earlier removal of the splint may inadvertently increase mobility in transplanted teeth in our young patients. This is even more important in the donors with short roots and in cases where the space between bony walls and transplant roots is wide. The gingival flap was adapted closely and neatly with sutures onto the neck of the transplanted tooth. This seal should be as water-tight as possible. Periodontal dressing was applied to protect the transplant against infection during the first 2 to 3 days in the wound healing. This dressing was removed at about 3 to 4 days after surgery. Antibiotic and 0.2% chlorhexidine mouthwash were prescribed for one week.

A radiograph was taken immediately after splinting to evaluate the new position of transplanted tooth and as baseline record. Postoperative assessments and intervals of followup are as in Table 1. The parameters listed in the table were used for evaluation of the outcomes.

## 3. Discussion

The primary purpose of autotransplantation is to recreate a functional tooth. Losing an important tooth like a permanent maxillary incisor in a young patient usually means lifelong prosthesis or orthodontic closure with compromised aesthetic outcome. The option of autotransplantation of ectopic maxillary incisor may be considered when orthodontic traction into alignment is not possible and this option should be explored prior to surgical removal of the tooth.

The major advantage of autotransplantation in a young patient is the induction of alveolar growth which allows eruption process to occur [2, 7, 8]. The patient regains a proprioceptive feeling in the transplanted teeth, with normal periodontal healing, allowing a natural feel during chewing [8–11]. Orthodontic treatment can be implemented if required [2, 6, 9] as opposed to other prosthetic replacements such as osseointegrated implant.

Prognosis of autotransplantation is good if the following happens:

1. Patient is healthy, compliant, and able to maintain a good oral hygiene [8, 10].
2. The ideal stage of donor tooth is when the root has 3/4 of its development [2, 12] and an open apex of more than 1 mm [2, 6, 13].
3. The ideal receptor alveolus has sufficient height and width to shelter the donor tooth [14, 15].
4. Surgical technique is meticulous and atraumatic [6, 9, 14] with reduced extra-alveolar time [2, 8, 14].This is to preserve the periodontal ligament and maintain the Hertwig's Epithelial Sheath so that root development is not compromised.

The most decisive factor for periodontal healing after surgical transplantation is the presence and viability of the periodontal ligament of the tooth [2, 4, 6]. Reports on success rate of autotransplantation vary from 79% to 100% in the long term cohort study [11, 14] depending on the duration of followup and criteria of success. The transplants are recorded as successful if there are positive vitality response, normal periodontium, and normal root development [14] as well as the absence of pathology, ankylosis, and decreased root length (crown to root ratio > 1) [11].

During review, continued root development was observed in Cases 2 and 3 where the initial stages of root development were 1/4 root formed and 2/3 root formed, respectively. In immature teeth where the apex is open, pulp regeneration and revascularization are expected to occur. Thus, root canal therapy is not indicated unless complications develop. Even in Case 1 where the apical ends were almost complete or just completed, chances of pulpal healing were high due to young and large pulp. Extra-alveolar time was kept as minimal as possible, between 0 to 5 seconds, in order to maximize success.

The most common complications of autotransplantation are ankylosis and inflammatory root resorption. None of the transplanted teeth suffered from these complications from strict adherence to the suggested technique. Normal periodontium and absence of pathology were observed in all cases. All patients reported no discomfort associated with the transplanted teeth and perceived the transplants as not different from their other teeth. Should these complications develop, autotransplantation may still preserve bone volume in a young patient for future implant procedures [16].

## 4. Conclusion

Autotransplantation of ectopically positioned permanent maxillary incisors is an alternative to any other options and should be considered when making a treatment plan in our young patients. Successful tooth autotransplantation offers improved aesthetics, dentofacial development, arch integrity, and normal function.

## Figures and Tables

**Table 1:** Postoperative assessment of autotransplantation of teeth.

The following parameters are to be assessed and recorded during review appointments	
Clinical	Normal value
(i) Recession (measured to the cement-enamel junction or CEJ)	(i) No recession
(ii) Tooth mobility (grades 0–3)	(ii) Grade 0 or physiological mobility
(iii) Periodontal pockets depth (only done post-op > 6 months)	(iii) Pocket 3 mm or less
(iv) Bleeding on probing (presence or absence)	(iv) No bleeding
(v) Percussion test (tenderness and sound)	(v) No tenderness and normal sound
(vi) Colour of crown	(vi) Normal colour
(vii) Vitality testing (electric pulp and cold test)	(vii) Positive

Radiographic	Normal value
(i) Root growth	(i) Continuation of root growth
(ii) Dentinal walls of root	(ii) Thickening of dentinal walls of root
(iii) Periapical radiolucency	(iii) Resolving periapical radiolucency
(iv) Lamina dura	(iv) Presence of lamina dura
(v) Pulp obliteration	(v) No obliteration

*Intervals of follow-up*	

Review dates are given as follows:	
(i) 1 week post-op: review splint and oral hygiene	
(ii) 2 weeks post-op: review splint and oral hygiene	
(iii) 1 month and 2 months post-op: remove splint and carry out clinical and radiographic assessment	
(iv) 4 months, 6 months, 1 year post-op, and yearly: carry out clinical and radiographic assessment	

## References

[1] Yaqoob O., O'Neill J., Gregg T., Noar J., Cobourne M., Morris D. Management of unerupted maxillary incisors. https://www.rcseng.ac.uk/dental-faculties/fds/publications-guidelines/clinical-guidelines/.

[2] Tsukiboshi M. (2002). Autotransplantation of teeth: requirements for predictable success. *Dental Traumatology*.

[3] Kim S., Kim J., Song J. S., Choi H.-J., Choi B.-J., Kim S.-O. (2013). Continued root development of a surgically repositioned human incisor tooth germ. *Oral Surgery, Oral Medicine, Oral Pathology and Oral Radiology*.

[4] Agrait E. M., Levy D., Gil M., Singh G. D. (2003). Repositioning an inverted maxillary central incisor using a combination of replantation and orthodontic movement: a clinical case report. *Pediatric Dentistry*.

[5] Park J. H., Tai K., Hayashi D. (2010). Tooth autotransplantation as a treatment option: a review. *Journal of Clinical Pediatric Dentistry*.

[6] Andreasen J. O., Paulsen H. U., Yu Z., Bayer T., Schwartz O. (1990). A long-term study of 370 autotransplanted premolars. Part II. Tooth survival and pulp healing subsequent to transplantation. *European Journal of Orthodontics*.

[7] Paulsen H. U., Andreasen J. O. (1998). Eruption of premolars subsequent to autotransplantation. A longitudinal radiographic study. *European Journal of Orthodontics*.

[8] Kim E., Jung J.-Y., Cha I.-H., Kum K.-Y., Lee S.-J. (2005). Evaluation of the prognosis and causes of failure in 182 cases of autogenous tooth transplantation. *Oral Surgery, Oral Medicine, Oral Pathology, Oral Radiology and Endodontology*.

[9] Diaz J. A., Zaror C. E. (2014). Long-term evaluation and clinical outcomes of children with dental transplants in Temuco City, Chile. *European Journal of Paediatric Dentistry*.

[10] Aslan B. I., Üçüncü N., Doğan A. (2010). Long-term follow-up of a patient with multiple congenitally missing teeth treated with autotransplantation and orthodontics. *The Angle Orthodontist*.

[11] Czochrowska E. M., Stenvik A., Bjercke B., Zachrisson B. U. (2002). Outcome of tooth transplantation: survival and success rates 17–41 years posttreatment. *American Journal of Orthodontics and Dentofacial Orthopedics*.

[12] Jonsson T., Sigurdsson T. J. (2004). Autotransplantation of premolars to premolar sites. A long-term follow-up study of 40 consecutive patients. *American Journal of Orthodontics and Dentofacial Orthopedics*.

[13] Marques-Ferreira M., Rabaca-Botelho M.-F., Carvalho L., Oliveiros B., Palmeirao-Carrilho E.-V. (2011). Autogenous tooth autotransplantation: evaluation of pulp tissue regeneration. *Medicina Oral Patologia Oral y Cirugia Bucal*.

[14] Kvint S., Lindsten R., Magnusson A., Nilsson P., Bjerklin K. (2010). Autotransplantation of teeth in 215 patients—a follow-up study. *The Angle Orthodontist*.

[15] Northway W. (2002). Autogenic dental transplants. *American Journal of Orthodontics and Dentofacial Orthopedics*.

[16] Thomas S., Turner S. R., Sandy J. R. (1998). Autotransplantation of teeth: is there a role?. *British Journal of Orthodontics*.

